# Orthotic Device Use in Canine Patients: Owner Perception of Quality of Life for Owners and Patients

**DOI:** 10.3389/fvets.2021.709364

**Published:** 2021-11-04

**Authors:** Sera Lee, Theresa M. Wendland, Sangeeta Rao, Christianne Magee

**Affiliations:** ^1^Department of Biomedical Sciences, College of Veterinary Medicine and Biomedical Sciences, Colorado State University, Fort Collins, CO, United States; ^2^Department of Clinical Sciences, College of Veterinary Medicine and Biomedical Sciences, Colorado State University, Fort Collins, CO, United States

**Keywords:** orthotic, sports medicine, quality of life, client satisfaction, veterinary rehabilitation, canine orthopedics

## Abstract

Orthotic devices are an established treatment for neuromusculoskeletal disease in the human population. Orthoses are an emerging veterinary therapy due to limited practitioner experience, availability of devices, and published data from veterinary patient outcomes. Expanding client education and veterinary expertise in the application of orthoses may allow greater access and successful utilization of these devices to treat appendicular disease. While orthoses have the potential to improve quality of life for veterinary patients, consideration needs to be made for owner related factors with device use. Owner satisfaction and experience may greatly impact compliance with treatment recommendations; therefore, it is crucial that owner expectations are met. The purpose of the present study was to evaluate owner-reported outcomes of orthosis for canine patients and their owner's subjective responses about the shared pet/owner experience utilizing a promoter score. It was hypothesized that owner's impressions of their pet's experience with the orthotic device would influence owner perceptions of quality of life for both the owner and the pet, and these factors would impact the likelihood of the owner to recommend a veterinary orthosis to a friend. An anonymous online survey was sent to 136 clients of a single veterinary orthoses manufacturer. Fifty-six surveys were completed and included for analysis. The owner's reported quality of life was in agreement (*P* = 0.02) with reported pet quality of life. There was also a higher likelihood (*P* = 0.02) for the owner to recommend a veterinary orthotic device to a friend when owner perceptions of pet quality of life were positive as compared to negative or neutral. Willingness to recommend an experience to a friend is a reflection of satisfaction with the experience. The dependence of owner and pet quality of life should therefore guide therapeutic decisions for patient management and client communication to ensure that the orthosis experience is positive for both patient and owner.

## Introduction

Orthotic and prosthetic devices are an established treatment for human neuromusculoskeletal disease (1). Although the first “artificial leg” was described for a canine patient more than 50 years ago, orthoses are still considered an emerging or alternative therapy within the scope of veterinary medicine (2). Orthotic devices for veterinary patients are used for a variety of reasons, including preventing cast-related wounds, managing functional impairments in mobility and ambulation, and facilitating a return to a normal active lifestyle (3–5). Special consideration for device use is made for specific orthopedic conditions when surgery is not an option for the affected veterinary patient and/or client (e.g., financial constraints, concomitant diseases, increased anesthetic risk) (6, 7). Orthoses may additionally provide dynamic or adaptable post-operative coaptation that limits the need for long-term casting or bandaging (8). Many diseases, such as joint-level instabilities, tendinopathies, ligament injuries, and cranial cruciate ligament diseases (3, 9–12), which may have previously resulted in amputation or been medically unmanageable (8), can now be treated with orthotic devices, thus the demand for orthoses as a primary or adjunctive therapy is growing (2).

Pioneers of orthotics and external prosthetics in veterinary medicine have employed the expertise of human orthotists and prosthetists (8, 13) and have critically evaluated the clinical impact and outcome of these devices in veterinary patients (9, 14). Scientific study to develop evidence-based strategies for effective orthotic device use (3, 10–12, 15) cannot be denied as an absolute necessity; however, in the veterinary population it is the owner who must be sufficiently satisfied with the experience to continue treatment or recommend a treatment plan to other owners. The study of client satisfaction has contributed to other areas of veterinary medicine (10, 14, 16–18) and increasing our understanding of how veterinary patients are managed within the home environment can bring additional insight to the success of orthoses as treatment plans outside of the clinical setting. Therefore, analyses of owner satisfaction will contribute to sophistication of orthotic device use in veterinary medicine.

Although treatment success can be determined any number of ways, a consideration of the pet's quality of life (QOL) takes precedence when assessing elective therapies (19, 20). The purpose of most treatment plans involving an orthosis is to improve the QOL of the veterinary patient, but consideration must also be made for the impact a treatment may have on the owner's QOL and their burden as a caregiver. In veterinary medicine, the owner experiences the treatment process alongside their pet; therefore, it is crucial that both the patient's and the owner's needs and expectations are met. In human medicine, net promoter scores or “Friends and Family Tests” have been used to evaluate patient satisfaction with a medical procedure or hospital experience (21–24). While promotion scores have been used to assess client satisfaction from veterinary practice performance (25), a promoter score has not been used to evaluate a veterinary treatment modality. A primary aim of the study reported herein was to use a promoter score to quantitatively evaluate the shared orthosis experience by asking how likely the owner was to a recommend a veterinary orthosis to a friend. We hypothesized that the owner's impression of the pet's experience with the orthosis would influence owner perceptions about quality of life (QOL) for both the owner and the pet, and these factors would impact the orthosis experience promotion score. Owner-reported patient outcomes that may contribute to owner perceptions about pet QOL, including weight loss, ambulatory ability, and device complications, were used to determine if these objective factors impacted the owner's subjective responses for QOL or promotion score. Demographic factors of the owner were collected to provide descriptive information of the client base seeking orthosis treatment in the surveyed population.

## Materials and Methods

Clients of a veterinary orthotic device and prosthetic manufacturing company who had previously agreed to participate in follow-up study were selected to limit variability in device manufacture. Owners of canine patients who had used a veterinary orthosis prescribed by clinicians of a university veterinary teaching hospital were selected to limit variability in prescription and optimize sample size since canids are the most common species to receive devices from this company. All orthotic devices prescribed for this population of patients were of a custom hard shell hinged type to treat distal limb pathologies up to the level of the elbow joint for the thoracic limbs or up to and including the level of the stifle joint for the pelvic limbs. Email addresses of owners with only one canine patient per household were provided by the company but no other involvement in study design or data analysis was provided by the device manufacturer. Clients were emailed a letter by the first author describing the purpose of the study, the assurance that the survey would be anonymous, the choice for the owners to not participate in the survey, followed by a link to a third-party website where the survey had been created and hosted (SurveyGizmo). The survey was available for a total of 5 weeks between December 2017 and January 2018. A follow-up email was sent 3 weeks after the initial email to remind clients to participate in the study if they had yet to participate.

The survey (Supplementary Material 1) consisted of 20 questions and took approximately 5–7 min to complete. Questions were intended to gain information about the pet and orthotic device (e.g., species of the pet, age of the pet, weight of the pet at time of orthosis fitting, identification of limbs fitted with an orthosis, the orthopedic issue that necessitated the orthosis), objective factors that may have influenced owner perceived outcome (e.g., hours spent wearing the device, any device complications and/or their resolution, the pet's level of device tolerance and ability to ambulate while wearing the device, whether the pet partook in physical therapy or rehabilitation as part of their treatment, total cost of using the orthotic device), demographic information of the owner (e.g., age and income of the owner at the time they pursued treatment with an orthosis), owner perceptions of the device's impact on QOL for the owner and for the pet, owner likelihood to recommend an orthosis to a friend for their pet, and an open ended response for additional comments. QOL for either owner or pet was not quantified using a separate tool but was reported by the owner to obtain their subjective perceptions.

SurveyGizmo provided a summary of the anonymized survey response data (Supplementary Material 2). Some participants chose not to complete a question in the survey or answered in a way that was incompatible with accurate data collection. In either of these instances, the data for that question response was removed but all other valid survey question responses were retained. Owner responses for pet weight at the start of orthosis treatment and at the time of the survey were used to create a patient outcome for weight gain, weight loss, or maintained weight. The categorical data was described using counts and proportions and the continuous data was described using means and standard deviation. To analyze the agreement between owner-perceived QOL for the pet, negative and neutral responses on the survey in reference to QOL were combined to represent one collective representation against the solely positive responses. The continuous data was evaluated for normality assumption using Shapiro-Wilk statistics. If normality was not met, the data was analyzed using a non-parametric Wilcoxon 2 sample test. The Net Promoter Score® (NPS®) was determined by subtracting the percent detractors (extremely unlikely or unlikely to recommend) from the percent promoters (extremely likely or likely to recommend) (26). The categorical data was analyzed using Fisher's exact test due to <5 counts in some of the categories. Logistic regression analysis was used to calculate Odds Ratio to evaluate the likelihood of the outcome. For agreement between the outcome, such as QOL for the owners and pets, data was analyzed using Kappa statistic. A *p*-value of 0.05 was used to determine statistical significance. SAS v9.4 (SAS Institute Inc., Cary, NC) was used for all statistical analyses.

## Results

**Figure 1:**
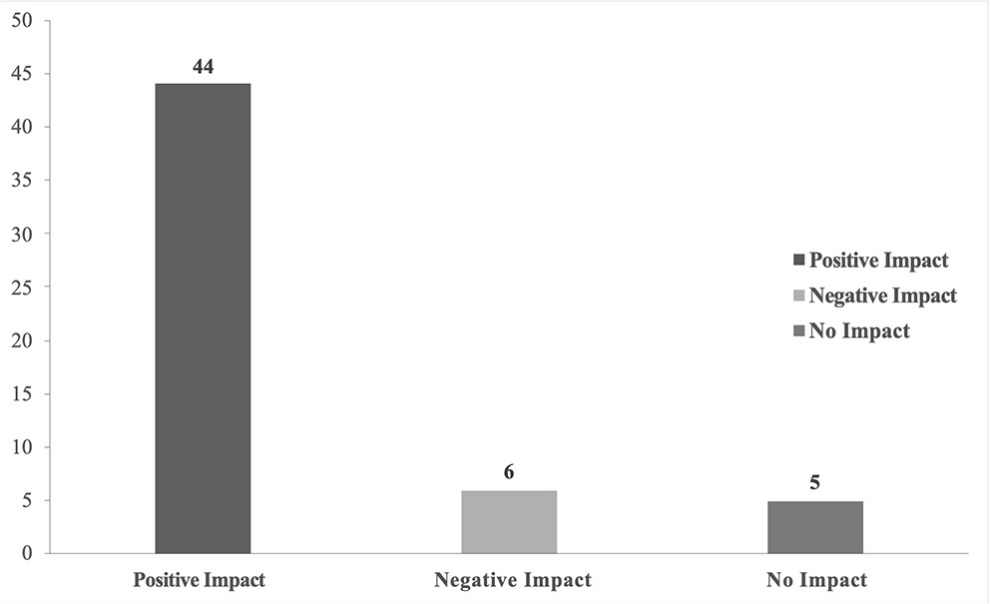
Impact of orthosis on pet's quality of life as perceived and reported by owner.

Of the 136 clients emailed a survey request, 56 surveys were completed, resulting in a 41.1% survey response rate. All survey responses confirmed canine patients and the distribution of limbs treated with an orthosis. Table 1 illustrates the application of an orthotic device within the responding client-patient population. The demographic distribution of device type in Table 2 illustrates the specific orthopedic issues that were treated with an orthosis. More than half of the responding owners (64%) reported complications during use of the orthotic device. Less than half of the owners (46%) reported using a form of physical therapy or rehabilitation as part of their pet's veterinary care during use of the orthotic device. More owners reported the device having a positive impact on their pet's QOL (78.6%) (Figure 1) than their own (53%) and the overall promoter score for a canine orthotic device was + 66.

**Table 1:** Distribution of limb that wore the orthosis.

**Limb**	***n* =**
Right thoracic	16
Right pelvic	23
Left thoracic	9
Left pelvic	19

**Table 2:** Distribution of orthopedic issues targeted by orthosis.

**Thoracic limb devices** ***n*** **=**	
Elbow brace	1
Carpal brace for carpal hyperextension	10
Carpal brace for reason other than carpal hyperextension	7
**Pelvic Limb devices** ***n*** **=**	
Stifle brace for cranial cruciate ligament injury	12
Tarsal brace for common calcaneal tendon injury	21
Tarsal brace for reason other than common calcaneal tendon injury	8
Brace targeting other region in pelvic/thoracic limb	3

**Figure 2:**
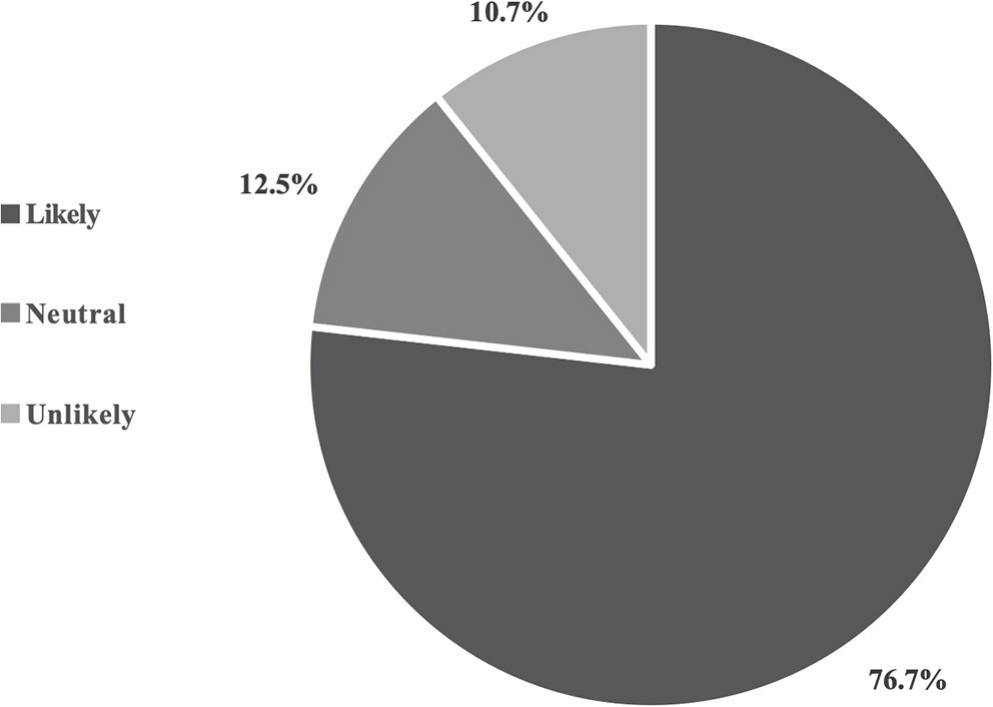
Owner's likeliness to recommend a veterinary orthosis to friends.

Out of 44 owners who reported the orthotic device as having a positive QOL impact on their pet, 29 of them also reported the orthosis as having positive QOL impact on their life as well. A significant agreement was noticed between the two QOL outcomes (*p*-value = 0.01). The likelihood to recommend an orthosis to a friend (Figure 2) was higher when the owner perceived pet QOL was positive when compared to when the owner perceived pet QOL was either negative or neutral (*p*-value = 0.02). The likelihood that the owner would recommend an orthosis to a friend was significantly higher when the owner reported a positive impact of the device on their QOL (*p*-value = 0.01). The device was described as worn to the full extent of the veterinarian's recommendation (compliance) by 83.6% of reporting owners. The number of hours that the device was reported to be worn by the patient was higher when there was compliance (*p*-value = 0.01). The device was reported as accepted or tolerated by 92.3% of the patients in this study. The likelihood of compliance with the treatment plan was higher (*p*-value = 0.02) when the device was tolerated by the patient. Pets that tolerated the orthotic device also wore it for longer periods of time compared to those that did not tolerate the device (*p*-value = 0.02).

**Figure 3:**
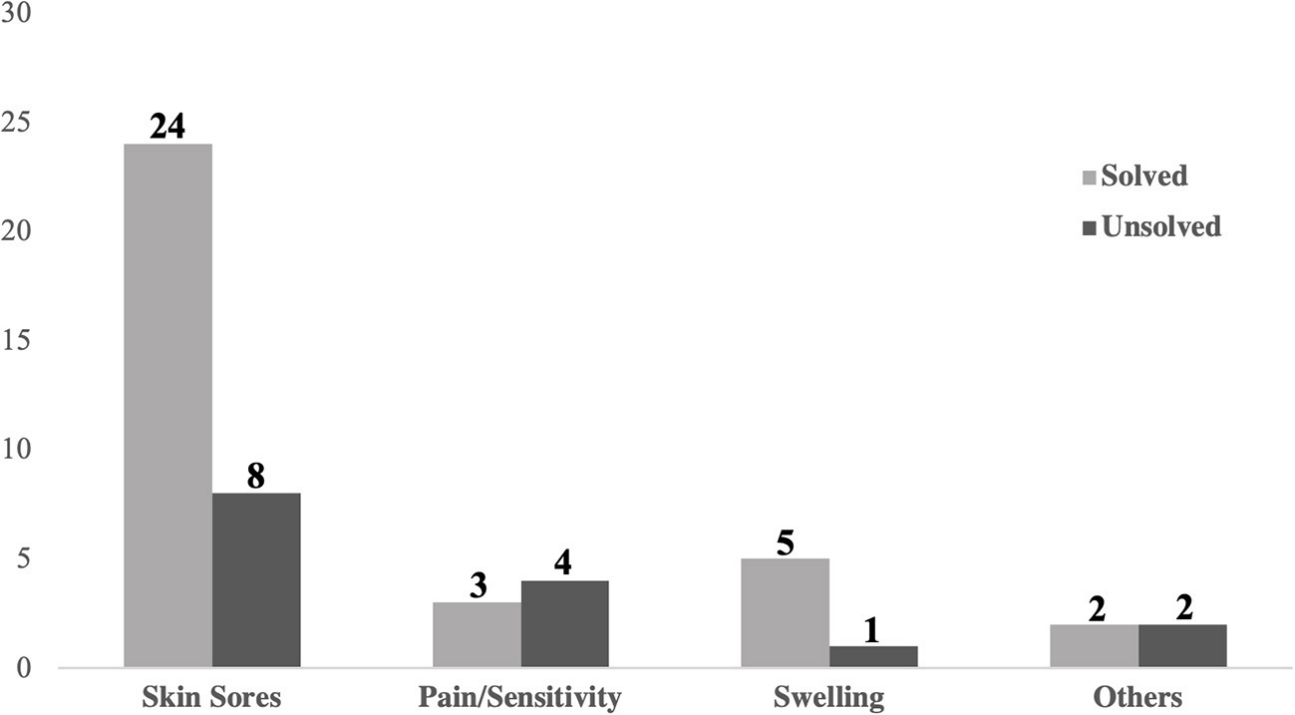
Owner reported resolution rate within device complication type.

**Figure 4:**
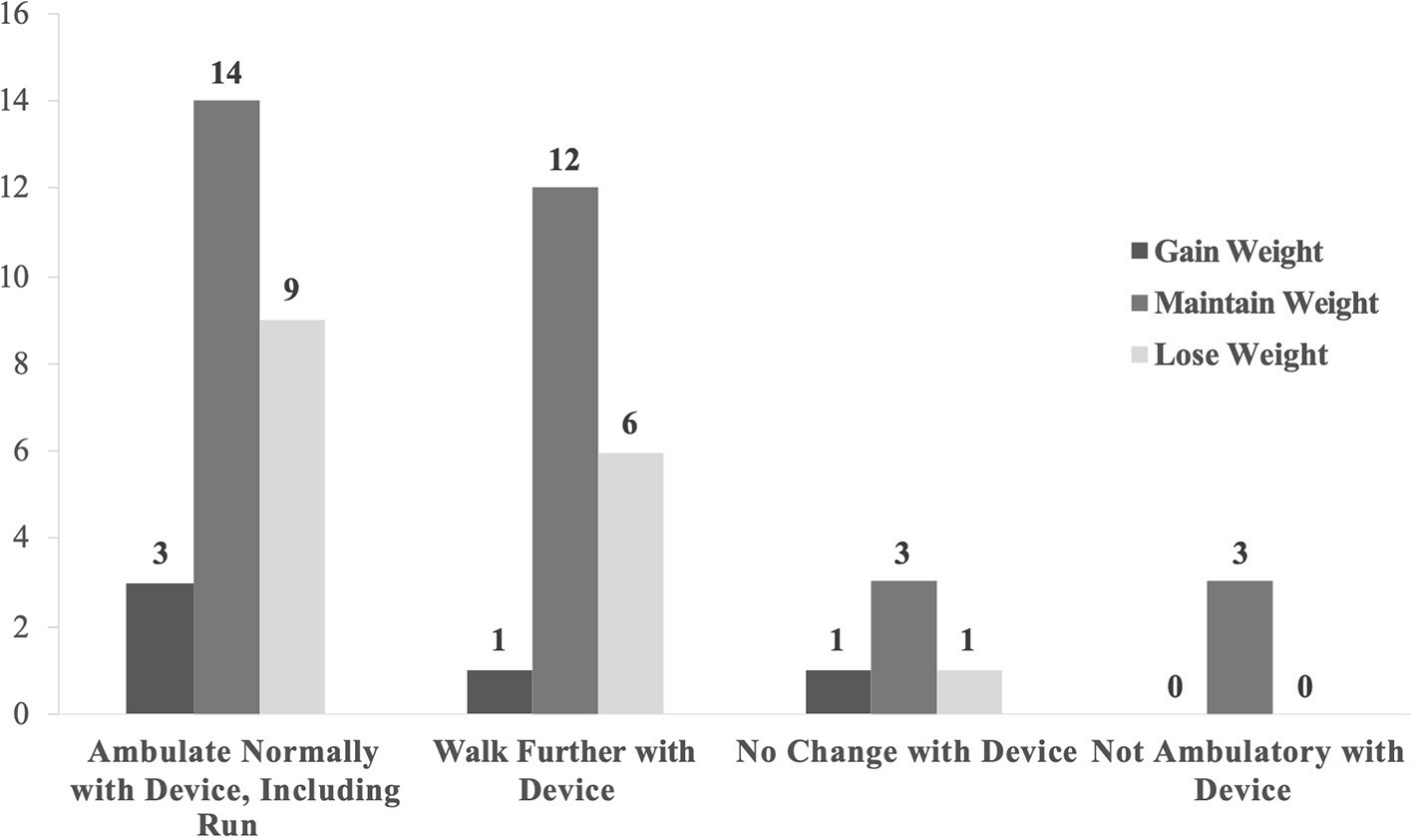
Owner reported patient weight change within ambulatory status.

The survey inquired about the owner's perception of objective outcomes that the pet experienced as a result of wearing the device. Several known orthosis complications were listed in the survey question as options to choose from (e.g., skin sores, pain/sensitivity, and swelling), with the opportunity for the owner to write in additional complications. Of the survey responses, skin complications were both the most common (*n* = 32) and the most frequently resolved (*n* = 24) complication at the time that the survey was completed (Figure 3). In this study, most owners (84.9%) reported improved ambulatory function as a result of the device and more than 90% of the canine patients either maintained or lost weight after initiating orthosis treatment (Figure 4). Ambulatory ability and changes in pet weight as reported by the owner were visually compared (Figure 4) with no statistically significant relationship between the two outcomes. None of the owner reported objective outcomes in this survey significantly changed their likelihood to report positive QOL outcomes for either themself or their pet, nor their likelihood to recommend orthosis to a friend.

**Figure 5:**
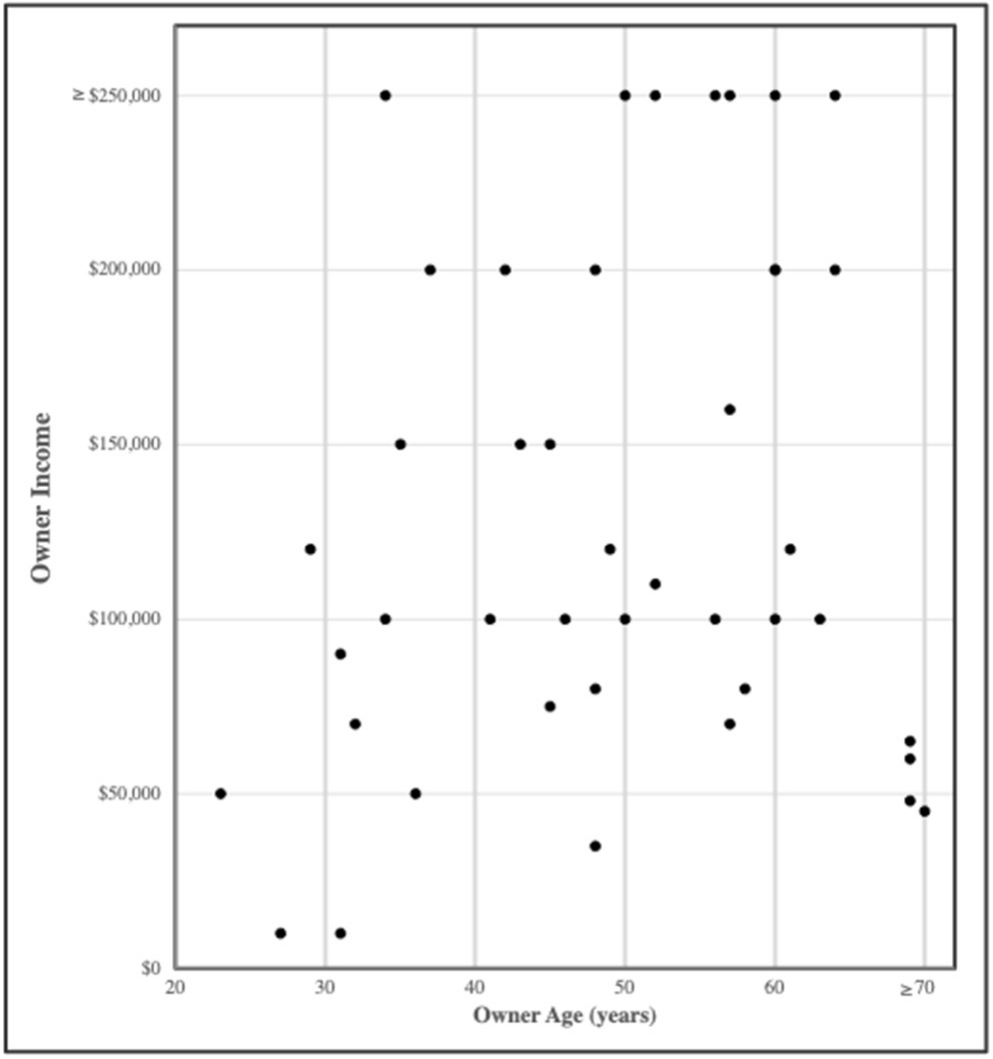
Owner reported age vs. income.

Of the 54 survey responses in which owner age was indicated, most (93%) of the respondents were at least 30 years old. Only 44 of 56 survey respondents provided information about their income. There was no significant relationship between age and income of the study participants (Figure 5). It was observed that of the 44 respondents who provided income information, 66% had an income > $100,000USD/year, whereas only 11.9% had income < $50,000USD/year.

## Discussion

Although veterinary orthotic devices are not a replacement for surgical treatment, there is evidence that they provide dynamic solutions to some orthopedic conditions (8, 27) and their veterinary application has resulted in non-surgical successes (10, 15, 28). This study assessed owner-reported orthotic device outcomes and used a promotion score to quantitatively evaluate the shared orthosis experience.

The clients contacted for this study had only one canine orthosis patient per household, indicating that a total of 56 canine patients are described by these survey results. In the survey questionnaire, data was collected by limb as well as by device, resulting in some survey respondents reporting information about multiple devices (*n* = 62) and/or treatment of multiple limbs (*n* = 67). These findings indicate that some patients had more than one orthotic device or more than one limb treated with an orthosis. The survey respondent's “additional comments” demonstrate that bilateral device use was the case for two patients with bilateral rupture of the gastrocnemius tendons. However, given the anonymous nature of the data, it is unknown whether the patients included in this study were using more than one orthotic device at a given time. If the client had more than one device on their pet at different periods of the pet's life, then this would indicate that they found sufficient resolution and satisfaction with their pet's first device to pursue a second orthotic device.

Analysis of customer's satisfaction, promoter scores, or online word-of-mouth behavior can be helpful to better understand a company's growth potential and/or customer loyalty (26, 29). In human healthcare, the patient is the “health-care consumer” and greater focus is being placed on satisfaction as an assessment of quality of care received (30, 31). The role of the caregiver cannot be underestimated in veterinary medicine and is a burgeoning area of study (32–35). To the author's knowledge there is no known relationship between caregiver burden and NPS® or similar “Friends and Family Tests” (36). In veterinary medicine, the consumer is the client responsible for the financial cost of treatment, making the healthcare decisions, and providing the care, which may directly impact owner perceptions about treatment outcomes and their likelihood to recommend the treatment. The NPS® can vary by industry, region, or characteristics of the survey respondents such as age, income level, or time with a service (37). In veterinary medicine, NPS® scores for veterinary practices vary with veterinary business publications reporting +30 as an average score for a practice that is “doing well” (38). In this study, the calculated promotion score for the canine orthosis was +66, which is a “good” score (39) and similar to the aggregated NPS® of +70 that has been reported for “medical devices” (40). In this context, the NPS® may provide a cumulative assessment of both the client and the patient's shared experience. Some veterinary market researchers suggest that “empowerment” of clients as active participants in their pet's care can result in a higher NPS® and that the age of the client can impact the efficacy of these strategies (38). A type of “placebo effect” has also been described for veterinary orthopedic patients which may contribute to owner perceptions about treatment outcomes (41). Much of veterinary orthopedic medicine depends on evidence from case reports and even the studies that have validated the role of the orthotic device use in canine patients may have some outcome bias (3–5, 7, 8, 11, 41). Nonetheless, one of the most complex aspects of the veterinarian-client-patient interaction is communication centered on the clinical decision-making process and satisfaction (38). It is possible that either caregiver burden or a caregiver placebo effect, resulting from a modification the owner's sense of involvement in their pet's wellness and/or a sense of owner empowerment, contributed to the owner's subjective assessment of the orthosis experience and the QOL and NPS® measures in the current study. Nonetheless, this study demonstrates a clear dependence of patient and owner QOL, which are then linked to the owner's satisfaction or promotion score with the orthosis experience. Veterinarians managing orthoses must use these relationships to differentiate between the owner's subjective measures of treatment success and validated, objective measures when assessing patient outcomes.

In this study, 86.3% of owners reported following the full extent of the orthosis device prescription, and less than half of the respondents utilized a form of physical therapy and/or rehabilitation (PT/R) for their pet. Like human physical therapy, veterinary PT/R can treat pain, improve circulation, range of motion, mobility, and coordination, reduce edema, and promote healing (8). An additional goal of a rehabilitation program for an orthotic device is to help integrate the orthosis into the lifestyle of both owner and patient. A well-designed physical rehabilitation treatment plan may accelerate recovery, while preventing future injuries or re-injuries, and permanent disabilities (42) and an accompanying prescription for PT/R is standard practice at the university teaching hospital where the orthosis was prescribed in this study. The owner rationale for the pet's participation, or lack thereof, in either the full extent of the device prescription and/or an accompanying PT/R prescription was not pursued as part of this study. In theory, evidence-based orthosis and/or PT/R prescriptions produce the desired results when followed to the full extent of the treatment plan, therefore, greater compliance with the prescribed treatment plan could improve patient outcomes. Despite more than half of the patients not participating in PT/R, owners were sufficiently pleased with the experience to recommend orthosis. Further study to understand the impact of caregiver burden on device compliance and pursuit of PT/R may help clinicians design orthosis and PT/R prescriptions that further improve both patient outcomes and the shared experience for both pet and owner.

The survey asked owners to report complications the pet experienced while wearing the device. Skin sores in particular are a common complication of veterinary orthotic and prosthetic devices (10, 43–45). With device modification and/or proper wound management sores can often be resolved, but are one of the primary reasons that veterinary oversight of orthotic use is recommended. In this study, skin sores accounted for more than half of the unresolved complications (Figure 3). Despite the overall frequency of complications, most owners reported that device use had a positive impact on their pet's life and were still likely to recommend the use of an orthosis to a friend. Due to the design of the survey, it was not possible to analyze which complications were more common, or which complications had a higher likelihood of resolving, based on the size, weight, body condition, and breed of patient. Future research to analyze the correlation and impact of patient variability with complications and/or outcomes would be ideal.

Owner satisfaction was considered with regards to contentment the owner experienced during and following the usage of the orthotic device for their pet in two retrospective studies analyzing the use of orthotic and prosthetic devices (10, 43). An owner's willingness to recommend an orthotic device as a form of treatment, an orthotic fabricating company, or the veterinarian who prescribed the orthotic device are all aspects in the promotion score that were not individually assessed. All the owners who were contacted for this study had at one time been clients of a single orthotic device company and a single group of clinicians; however, veterinary care may have been provided by other clinicians throughout the period of device use. The interaction of the client with the veterinary team providing orthosis support may have varied by demographic factors. As the age of the veterinary workforce changes (46) some veterinary market researchers differentiate clients by generational age and suggest different strategies for engaging Millennials (participants age 23–36 in this study) vs. Boomers (ages 53–71 in this study) to find value in customer service and patient care (38). Interestingly, of the clients who answered the income question in this study, over half of the respondents reported at least a $100,000/year income. It should be noted that this population represent less than one-third of the 136 clients initially contacted, but this finding suggests that the process of receiving an orthotic device, which is often paired with a specialty examination and diagnostic imaging such as CT, ultrasound, and/or radiographs, may be aided by having a higher than average income. Furthermore, of the 42/56 survey responses in which age was indicated, the majority of respondents were at least 30 years old, suggesting that they are more of the GenX and Boomer generations. The demographic data in this study suggests that individuals who elect treatment with an orthosis are most likely older than 30 years and of an upper middle-class socioeconomic status, which makes them more likely to have a graduate or professional degree (47). However, it must be noted that the surveyed population was from a referral veterinary hospital likely resulting in inherent population bias. Nonetheless, the demographic data may have influenced the provided promotion score and future studies may consider including the demographic data in their NPS® analysis.

The survey tool used in this study used few open response questions to limit the length of the survey and emphasize the collection of quantitative data that was considered the priority in the study design. The survey tool also did not inquire about the specific dog breed of the veterinary patient. As a result, parameters (e.g., complications, complication rate) that may be impacted by patient size (ie. toy, small, medium large, giant breeds) were not analyzed. In addition, the survey did not inquire about the general body condition and/or level of obesity of each patient. Multiple studies have shown that owners often inaccurately misperceive where on the body condition score scale their dog is represented (48, 49). Thus, asking clients to recall the pet's body condition score in the survey may have led to inaccurate analyses of the role of pet obesity in the analyses. Instead, pet weight outcome was determined using owner reported pet weights. Increased patient body weight has long been known to negatively impact outcomes following non-surgical management of canine orthopedic issues (15) and weight management is frequently included in the overall treatment plan for dogs with orthopedic disease. In this study, most of the patients either lost or maintained weight after initiating orthosis treatment. The strategies employed to prevent weight gain in these patients cannot be determined with the study design. However, the majority of patients in this study were able to walk further or ambulate more normally as a result of the device (Figure 4), presumably aiding in their weight management program. It is unknown if the animal was considered overweight at the time of orthosis prescription; presumably if 30.2% of the patients lost weight, then some of the patients had additional body mass to lose. Although owner estimates of pet body conditions score are often inaccurate (48), future studies could potentially include the general size of the dog (i.e., small, medium, large), weight management strategies, as well as body condition score so that these data could contribute to the analysis of clinical orthotic device success and client satisfaction.

Although the survey response rate in this study was similar to other orthotic device related client surveys (10, 43), the client population surveyed was intentionally limited to clients of a single hospital and orthosis manufacturer which may limit application of these findings. If designing a future survey-based study, mid-December to mid-January is a time of year known for a reduced response rate (47, 50) and distribution during another time of year may help increase the number of survey responses. Furthermore, in this study there was only one reminder email sent out 3 weeks following the initial email containing the survey link; the use of additional reminder emails and a longer response period may result in more survey responses. In some of the analyses for this study, the relatively small response pool limited the power of the statistical analysis that could be performed with the categorical data. Future studies may also focus groups to more specifically explore factors that influence the shared owner and pet orthosis experience. In addition, extrapolating cases from one veterinary orthotic manufacturer and one institution may have resulted in population selection bias based on institutional recommendation. This limitation, however, may have controlled for variation in clinician case management. Another limitation is the inherent voluntary nature of the survey rather than a required event as part of the treatment program. Despite these limitations, the data that was obtained from the surveyed population demonstrates that canine owners perceive orthoses to have a positive impact on QOL for both them and their pets such that they would recommend orthosis to a friend regardless of the other reported treatment outcome factors.

It should be noted that many veterinary orthotic and prosthetic devices are produced per a veterinarian's prescription (8). Based on the evidence from the case log of the veterinary orthotic and prosthetic company in the present study, veterinarians prescribe orthotic devices more frequently than prosthetic devices. Literature supporting the use of either orthotic or prosthetic devices is limited, but the clinical application of orthotic devices is more varied and pertinent to a wider range of orthopedic conditions than prosthetic solutions. Orthotic devices have garnered increasing interest, value, and significance in the field of veterinary medicine due to their wide range of applicability (2). With the help of human orthotist prosthetist expertise (8), the field of veterinary medicine is increasingly understanding the complexity and intricate design of quadruped mobility and biomechanics and how the application of orthotic devices can help to better maintain a quadruped lifestyle if at all possible for certain patients (27). This report of the shared pet and client experience from the owner perspective will aid in understanding how orthotics are of benefit to veterinary medicine.

## Conclusion

Orthotic devices are gaining more mainstream integration into veterinary medicine. In addition to understanding the clinical viewpoint of orthotics in veterinary medicine, the importance of analyzing the owner's subjective experience with orthosis treatment should not be underestimated. Being able to gain insight into the demographics of those who use an orthosis for their pet may aid in the development of client communication strategies associated with orthosis treatment programs. This study was intended to provide the veterinary field with owner's perceptions of the shared pet and owner orthosis experience. It was hypothesized that owner's impressions of their pet's experience with a veterinary orthosis, including outcome factors such as device complications, mobility in the device, and device tolerance, would influence owner perceptions about QOL for both the pet and the owner. From this study one can conclude that the surveyed owner population perceived orthotic device use to have a positive impact on both their QOL and that of their canine patient. In order to quantitatively assess the shared pet and owner experience with the orthosis, it was assumed that if the owner was willing to recommend pursuing a canine orthosis to a friend, then this recommendation indicated that their shared experience had resulted in positive outcomes for the patient and owner; these expectations under the original hypothesis were met for pet and owner QOL. The remaining outcome factors (e.g., complications, mobility, etc.) did not influence either owner perceptions of QOL or the promoter score. The dependence of owner and pet quality of life and the associated satisfaction with the orthosis experience should guide veterinary care providers and orthosis manufacturers to evaluate their approach to ensure that the orthosis experience is positive for both patient and owner.

## Data Availability Statement

The original contributions presented in the study are included in the article/Supplementary Materials, further inquiries can be directed to the corresponding author/s.

## Ethics Statement

The studies involving human participants were reviewed and approved by Institutional Review Board - Colorado State University. The patients/participants provided their written informed consent to participate in this study.

## Author Contributions

SL, CM, and TW postulated the experimental design. SL performed work associated with this study. SL and CM prepared manuscript. SR performed statistical analysis. All authors reviewed manuscript upon final submission.

## Funding

Funding was provided by a Thesis Improvement Grant to Sera Lee from the Colorado State University Honors Program.

## Conflict of Interest

The authors declare that the research was conducted in the absence of any commercial or financial relationships that could be construed as a potential conflict of interest.

## Publisher's Note

All claims expressed in this article are solely those of the authors and do not necessarily represent those of their affiliated organizations, or those of the publisher, the editors and the reviewers. Any product that may be evaluated in this article, or claim that may be made by its manufacturer, is not guaranteed or endorsed by the publisher.
