# Supplementary material for: Orthotic Device Use in Canine Patients: Owner Perception of Quality of Life for Owners and Patients
Source: Front Vet Sci. 2021 Nov 4;8:709364. doi: 10.3389/fvets.2021.709364 (PMC8600258; doi:10.3389/fvets.2021.709364)
Supplement: Supplementary file 2 [file Data_Sheet_1.PDF]

Thank you for taking your time and participating in this survey. This survey is completely anonymous and should take no more than five to seven minutes of your time.

1. What species is your pet?
   ____Dog
   ____Cat
   ____Other ___________________

2. How old was your pet at the initial application of the orthotic device?
   _____ years

3. How old is your pet now?
   ____ years
   ____Pet is deceased
   ____Age at time of death

4. Which limb(s) does your pet wear the orthotic/prosthetic device(s)? Check all that apply to your pet
   ____Right forelimb
   ____Right hindlimb
   ____Left forelimb
   ____Left hindlimb

5. Which of the devices below does your pet wear?
   ____Knee brace for CCL (ACL) injury
   ____Ankle (tarsal) brace for an Achilles tendon injury
   ____Ankle (tarsal) brace for other reason
   ____Wrist (carpal) brace for carpal hyperextension
   ____Wrist (carpal) brace for other reasons
   ____Elbow brace
   ____Prosthesis for congenital deformity
   ____Prosthesis for elective amputation
   ____Prosthesis for unknown trauma
   ____Other (please describe________)

6. Was the device used to the full extent of the veterinarian's recommendation?
   ____Yes
   ____No
   ____If no, please explain the change in timeline and reason for that change:

7. On average, how many hours each day does your pet spend wearing the orthotic/prosthetic device?
   ____ hours

8. How much did your pet weigh the time of device prescription?
   ____ lbs

9. How much does your pet weigh now?
 ____ lbs

10. Did your pet have any complications due to the use of the orthotic device? Please check all that apply:
 ____Skin sores
 ____Swelling
 ____Pain/sensitivity
 ____Other, Please describe:

11. Did the complications resolve?
 ____Yes
 ____No, please explain: _________

12. Please rate your pet's level of tolerance while wearing the orthotic device.
 ____Did not accept device
 ____Accepted device but would chew/lick/remove device
 ____Accepted device while being supervised
 ____Accepted device with no attempt to chew/lick/remove device
 ____Other, Please describe:

13. Please select which explanation best matches your pet's ability to ambulate (move around) while wearing the orthotic device:
 ____My pet was not able to ambulate while wearing the device
 ____My pet was able to walk further while wearing device than without the device
 ____My pet was able to ambulate normally with device, including run
 ____The device resulted in no change in my pets ability to ambulate
 ____Other, Please describe:

14. Has rehabilitation or physical therapy been part of your pet's veterinary care?
 ____Yes
 ____No

15. How has your pet's quality or duration of life been impacted by the orthotic device.
 ____very positive impact
 ____somewhat positive impact
 ____no impact
 ____somewhat negative impact
 ____very negative impact

16. Which of the following statements best describes how the orthotic device for your pet has impacted *your* quality of life. Please select any statement that resonates with you.
    a. My pet wearing the device has not changed my quality of life - no impact
    b. The device has allowed my pet to return to more normal function, improving my quality of life - positive
    c. Getting the device to fit correctly has been a challenge for my quality of life - negative
    d. The device wear schedule has been detrimental to my quality of life - negative
    e. The device wear schedule has had no impact on my quality of life - no impact

17. How likely would you to choose to have your pet use an orthotic device and/or recommend an orthotic device to a friend for their pet?
    ____extremely unlikely
    ____unlikely
    ____neutral
    ____likely
    ____extremely likely

18. What has been the total cost of your pet's orthotic device? This price should include the initial examination, any procedures completed to prepare for the application of the device, to final fitting of the device.
    $____

19. How old were you at the time of the decision to pursue an orthotic device for your pet?
    ____ years

20. What was the average annual income of your household at the time of the decision to pursue an orthotic device for your pet?
    $ ____

Please provide any additional comments here: ________

Thank you for taking the time to complete this survey.
